# What is known about the development of relational competence in the mental health education of healthcare professionals – a scoping review

**DOI:** 10.1186/s12909-025-08353-7

**Published:** 2025-12-03

**Authors:** Lise Saestad Beyene, Miriam Dubland Vikman, Geir Tarje Fugleberg Bruaset, Marit Helene Hem, Elisabeth Andersen, Unn Elisabeth Hammervold

**Affiliations:** 1 Faculty of Health Science, University of Stavanger, Kjell Arholms gate 41, Stavanger, 4021 Norway; 2 VID Specialized University, Misjonsmarka 12, Stavanger, 4024 Norway; 3 Faculty of Health Studies, VID Specialized University, Diakonveien 14-18, Oslo, 0370 Norway; 4 Center for Mental Health and Substance Use, Lovisenberg Diaconal Hospital Ltd, Oslo, 0440 Norway

**Keywords:** Mental health education of healthcare professionals, Relational competence, Scoping review, Students, Therapeutic relationships

## Abstract

**Background:**

Therapeutic relationships with persons with mental health challenges are central to healthcare professionals’ work in mental healthcare. In the context of mental healthcare, healthcare professionals often work with persons who have challenges related to their emotional lives, which may be particularly challenging in relational work. This places high demands on the relational competence of healthcare professionals. An overview of pedagogical strategies to promote relational competence in mental health education of healthcare professionals is lacking and more knowledge is needed in this field. The aim of this study was to examine the body of knowledge on how the development of relational competence can be promoted and hampered in mental health education of healthcare professionals, as well as to identify knowledge gaps in this field.

**Methods:**

A scoping review was conducted with systematic searches in Medline, PsychInfo, Cinahl, Eric, Embase, and British Nursing Index, as well as hand searches in the reference lists of the included studies.

**Results:**

Forty scientific studies were included in this review in which qualitative methods (*n* = 18), quantitative methods (*n* = 16), and mixed methods (*n* = 6) were employed. The results show interventions that promote and hamper the development of relational competence in the mental health education of healthcare professionals. Learning interventions were organised into two themes. The first, individual active participation, describes promoting interventions and includes the sub-themes mapping training needs, learning through reflection, learning through active engagement in the classroom, learning in real-life situations, and learning through knowledge of lived experience. The second theme, receiving knowledge passively, describes hampering factors, which includes the sub-theme of learning through an explanatory theoretical approach.

**Conclusion:**

Learning interventions focusing on students’ individual active participation seem to promote the development of relational competence. Receiving knowledge passively is found to hinder the development of relational competence.

## Introduction

 Therapeutic relationships with persons with mental health challenges are central to health professionals’ practice in the context of mental healthcare. Relational work is essential for recovery and health-promoting processes [1], and the quality of the relationship between the person with mental health challenges and the health professional is considered decisive [2]. To achieve recovery, persons with mental health challenges and health professionals must form therapeutic relationships as part of the health promotion process. For up to 30% of those with mental health challenges, recovery depends on health professionals’ ability to achieve therapeutic relationships that provide a basis for health promotional processes [3]. Consequently, relational attributes are important, as research shows that some health professionals are better than others at forming effective relationships with persons with mental health challenges [4]. A health professional who has strong relational competence can, through professional knowledge and insight, interact with someone with mental health challenges in a way that may promote the person’s mental health [1]. Despite its acknowledged importance, the concept of relational competence lacks a unified definition. Relational competence addresses how health professionals exercise their professional knowledge and skills in human interaction, who they are as persons, how attitudes are expressed, and how they appear when meeting others [5]. Relational competence further implies “having a genuine interest in understanding the patient, engaging in reciprocal interaction with the patient, meeting the patient so that they feel acknowledged and having the ability to self-reflect and self-regulate’ ([6], p.17).

Health professionals in mental healthcare often work with persons with severe problems in their emotional lives, which may particularly challenge relational work. In this relationship, the health professional must respond to the person’s destructive patterns and exercise relational and emotional skills such as empathy, problem-solving, conveying hope and faith for the future, and having self-awareness and self-control. This requires the ability to reflect on, react to, and understand different situations professionally [5]. Emotional intelligence underpins the health professional’s relational competence. Emotional intelligence implies an ability to process emotional information based on how one perceives, understands, expresses, regulates, and handles emotions. It involves the mental capacity to be aware of one’s attitude towards one’s own emotions and to distinguish between emotions and having good strategies for emotion regulation [7–9].

Research on therapeutic relationships between persons with mental health challenges and health professionals indicates factors deemed ineffective for the persons with mental health challenges’ recovery. This concerns, among other things, health professionals acting in ways perceived as rude, unprofessional, and inappropriate in communicational terms and who meet persons with mental health challenges with prejudice [10]. Donati [11] applies the concept of “touch and go” to describe health professionals’ distancing behaviours that create difficulty in establishing genuine, deeper relationships with persons with mental health challenges.

Students enrolled in health-related education programs typically belong to disciplines such as nursing, medicine, psychology, physiotherapy, occupational therapy, social work, and social educator. When aiming to promote relational competence in mental health education of healthcare professionals, it is important to strengthen students’ self-awareness, emotional competence, and interpersonal skills. Teaching emotional skills requires an environment that values and exemplifies these skills. Students learn to identify, understand, regulate, and balance emotions by, for example, exploring and assessing emotional situations and reflecting on their own and other people’s interactions. Values inherent in emotional development should be modelled for students. Relational skills in the curriculum should be based on the value of empowering students to master highly emotionally charged situations. Here, they must learn to handle their own emotions and the emotions of others respectfully, competently, and safely [7, 12].

Research shows that nursing students experience anxiety towards persons with mental health challenges [13], and widespread misconceptions and stereotypes about mental illness are often found in educational settings [14]. As an example, the curriculum for nursing students appears to lack student skills preparation in showing empathy, active listening, communication, and self-appearance in clinical mental health work – all considered important for establishing and achieving therapeutic relationships [15]. A gap has been observed between expectations in the field of practice regarding health professionals’ relational competence after completing higher education and the focus on this competence in mental health education of healthcare professionals [16].

Two important milestones are central to teaching students in mental health work. The Bologna Declaration entailed a transformation of the education system into a common degree system for Europe [17] to achieve a more streamlined education offering that allows the comparison of educational programmes both nationally and internationally. A degree system was introduced and, consequently, established mental health education was discontinued, and new programmes were established. The new programmes were interprofessional and the content of study programmes changed in line with new policy guidelines for mental health services [18, 19]. These guidelines – the second milestone – introduced a shift in the mental health services from a medical paradigm focusing on disease to a humanistic paradigm characterised by health promotion and recovery. Central in those approaches are user participation, and collaboration where professionals’ relational competence is central [20, 21].

Teachers in higher education are responsible for ensuring a good connection between the understanding of relational competence and how to facilitate students’ development of relational competence. The development of relational competence implies learning, meaning that we connect something new with knowledge and experience we already have, either by adding knowledge to existing understanding (assimilative learning) or by restructuring established understanding (accommodative learning) [22]. Relational competence involves personal changes. Pedagogical methods for developing this competence must therefore be linked to accommodative learning. Central concepts include raising awareness, critical thinking, and reflection [22]. However, accommodative learning is insufficient for making personal changes in students. Understandings and opinions must be transformed within the student and his/her behaviour for the new attitudes to be integrated. Transformative learning challenges existing understandings and viewpoints. Through this process, new skills are cultivated within students, embedding themselves so profoundly that they naturally guide future actions. Learning is a holistic process of adaptation to the world that involves the whole person, including thinking, feeling, perception, and behaviour, and results from synergetic transactions between the individual and the environment [22]. In a learning process, one moves between being an actor and being an observer, and between concrete participation and analytical distance. This entails concrete experience, reflective observation, abstract conceptualisation, and active experimentation [22].

Understanding how to promote relational competence in higher education can help create more inclusive, supportive, and enriching learning environments. While higher education research often focuses on academic and cognitive factors, relational competence seems to be understudied. To our knowledge, no comprehensive literature reviews have been conducted on this topic. This underscores the potential value of a more thorough review of existing research in the field. An overview of pedagogical strategies to promote relational competence in mental health education of healthcare professionals is lacking, and more knowledge is needed in this field [16]. Consequently, insights into how educators can help students develop these skills are important.

### Aim and research question

This study aims to examine the body of knowledge on how the development of relational competence can be promoted and hampered in the mental health education of healthcare professionals. Additionally, it seeks to identify knowledge gaps in the field.

## Methods

We carried out a scoping review to examine the body of knowledge on relational competence in the mental health education of healthcare professionals. We combined the guidance of Peters et al. [23] for conducting scoping reviews, based on the *Joanna Briggs Institute Reviewers’ Manual* [24], and the stepwise stages developed by Arksey and O’Malley [25] as further enhanced by Levac et al. [26]. To transparently illustrate the screening and selection process, we used a PRISMA flow diagram to map the number of records identified, included, and excluded at each stage of the review [27].

Scoping reviews are considered particularly relevant for examining broader areas to summarise and disseminate research findings and also identify research gaps and recommend areas for future research [25]. As initial searches revealed differences in the body of literature on the development of relational competence in terms of design, method, and context, we found a scoping review appropriate. Scoping reviews allow for a broad approach to the topic of interest and inclusion of studies regardless of methodological design. In scoping reviews, a formal assessment of included studies is generally not performed [28].

### Search strategy

#### Stage 1: identifying the research question

The search strategy is based on Arksey and O’Malley’s [25] methodological framework. In stage 1, the entire research group developed the aim and research question informed by preliminary literature searches on relational competence in the mental health education of healthcare professionals.

#### Stage 2: identifying relevant studies

In stage 2, actual studies were identified based on the research question and the aim of the study. The search terms were derived in line with Population-Concept-Context (PCC) components [23] and discussed among all reviewers before the searches. Peters et al. [23] recommend the PCC concept as a guide aiming to construct a clear and meaningful title and inclusion criteria for a scoping review (Table 1)

**Table 1:** Overview of the Population-Concept-Context components

Population	Concept	Context
Students enrolled in health and social care, education programs (e.g., nursing, psychology, social work) whose learning or training involves mental health-related topic	How development of relational competence are promoted or hampered	Universities and university colleges in Western countries

Western countries were selected as the contextual focus due to their well-established educational systems, structured mental health services, and extensive research traditions in mental health treatment and care including relational competence. This choice enables meaningful comparison and potential transferability of findings across similar socio-cultural and institutional settings.

The search string design was developed with the support of a qualified librarian, who performed test searches based on the terms and keywords elaborated. The search string was further developed in collaboration with LSB and UEH. Systematic searches were performed on 22 June 2022 in Medline, PsychInfo, Cinahl, Eric, Embase, and the British Nursing Index to identify relevant papers published between 1 January 2002 and 22 June 2022. The systematic searches were updated on 22 October 2023. We considered the chosen publication period appropriate based on milestones described in the education system and mental health services. We later searched the included publications’ reference lists for pertinent publications. In accordance with Arksey and O’Malley’s framework (2005), we consulted two academic experts who have published peer-reviewed research on relational competence. These persons were considered relevant stakeholders due to their subject-matter expertise and familiarity with the existing literature. We asked whether they could suggest additional pertinent publications, but this process did not yield further sources.

#### Stage 3: study selection

In Stage 3, we conducted the study selection based on publications identified through systematic searches (Table 2).

**Table 2:** Example search in Cinahl – Ebsco host 28.06.22

#	Searches	Results
S1	((relation* OR interpersonal OR inter-personal OR social) W0 (competenc* OR skill*)) N3 (develop* OR education OR training OR improve* OR improving OR teaching OR learning)	3490
S2	(“emotional intelligence” OR empath*) N3 (develop* OR education OR training OR improve* OR improving OR teaching OR learning)	1827
S3	S1 OR S2	5274
S4	((nurs* AND (universit* OR college* OR education OR student* OR graduate*)) OR (MH “Education, Health Sciences”) OR (MH “Education, Nursing+”) OR (MH “Schools, Health Occupations” OR (MH “Students, Health Occupations+”))	311,688
S5	S3 AND S4	1007
S6	mental OR psychiatr*	369,744
S7	S5 AND S6	137
S8	((MM “Professional-Patient Relations”) OR (MM “Professional-Client Relations”) OR (MM “Nurse-Patient Relations”) OR (MM “Interpersonal Relations”)) AND ((skills or competenc*) N3 (develop* OR education OR training or improve* or improving or teaching or learning))	1369
S9	S4 AND S8	388
S10	S9 not S5	303

The publications were retrieved and organised in the Endnote bibliographic management system, after removing duplicates, and then transferred to Rayyan (www.rayyan.ai), a web tool designed for working with different types of reviews and other knowledge synthesis projects. Titles and abstracts were blinded and double-screened in pairs by all six researchers. Discrepancies between pairs were resolved through discussion with a third researcher.

Full-text articles were then independently reviewed by all six researchers, working in newly formed pairs. During this process, the criteria for inclusion and exclusion were further refined. The exclusion criteria were as follows: (1) studies conducted in non-Western countries (2), Non-English language (3), incorrect publication type (4), focus on relational competence outside of mental health contexts, and (5) participants in studies who were graduated. Although formal critical appraisal is generally not recommended in scoping reviews [21, 23], we chose to critically read the included studies aiming to get impressions of the methodological quality. We did not exclude any publications based on this assessment, but several methodological weaknesses were identified. In the quasi-experimental studies, concerns included limited similarity between comparison groups, inconsistent treatment or care aside from the intervention, absence of control groups, and lack of repeated outcome measurements before and after the intervention. In some qualitative studies, the appropriateness of the chosen methodology was unclear, and descriptions of recruitment strategies were insufficiently linked to the research aims. Additionally, several studies lacked reflection on the relationship between researchers and participants. These issues suggest some risk of bias across the included literature. Inclusion and exclusion criteria for article selection are outlined in Table 3.

**Table 3:**
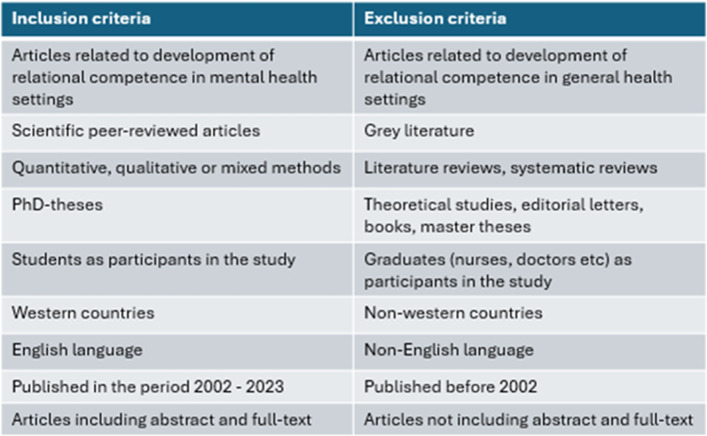
Overview of inclusion and exclusion criteria

#### Stage 4: charting the data

Stage 4 entailed charting data from the included studies by extracting and coding each article according to the following variables: authors, years of publication, intervention type, concepts, and key findings related to the review question. The data-extraction process was first conducted by LSB, who developed a charting form to record key information [28], and then controlled by UEH, who aimed to ensure that data related to the aim of the study and the research questions [23, 26]. The charting form, performed as a Word-document (part of Table 4), was shared and discussed with the other researchers.

**Table 4:** Synthesis of the results

Analytical themes	Descriptive themes	Intervention	Study
Individual active participation	Mapping training needs	Psychiatric/Mental Health Clinical Placement Survey (PMHCPS)	Julia-Sanchis et al. (2020) [29]
		Assessment for the inter-personal skills	Perry & Linsley (2006) [30]
	Learning through reflection	Action learning (including reflection)	Waugh et al. (2014) [31]
		Progressive journal prompts	Harrison & Fopma-Loy (2010) [32]
		Reflective groups	Gould & Masters (2004) [33]
	Learning through active engagement in the classroom	VR simulation	Traister (2023) [34]
		Simulation	Bee, Watkins & Barnes (2023) [35]
		Online game	Rodríguez-Ferrer et al. (2022a) [36]
		Online game	Rodríguez-Ferrer et al. (2022b) [37]
		Training and coaching GENOS model on Emotional Intelligence	Hurley et al. (2020) [38]
		Simulation	Baisden & Gray (2020) [39]
		Video game	Chen et al. (2018) [40]
		Simulation	Witt et al. (2018) [41]
		Simulation	Speeney et al. (2018) [42]
		Intentional Relationship Model (IRM) workshop/simulation	Hussain et al. (2018) [43]
		Role-playing activities	Campbell et al. (2017) [44]
		‘Talking Head’ video clips	Snelgrove et al. (2016) [45]
		Simulation and role-play	Fossen & Stoeckel (2016) [46]
		Standardized patients (simulation)	Webster (2014) [47]
		Simulation	Miles et al. (2014) [48]
		Role play	Lewis et al. (2013) [49]
		Simulation	Szpak & Kameg (2013) [50]
		The use of video	Stiberg et al. (2012) [51]
		Role play	Tiuraniemi et al. (2011) [52]
		Traditional classroom, employing experiential teaching modes	Hen & Goroshit (2011) [53]
		Simulation	Robinson- Smith (2009) [54]
		Classroom activities that included art, music, literature, and film to enhance the student understanding of mental illness	Jensen & Curtis (2008) [55]
	Learning in real-life situations	Clinical placement	Patterson et al. (2023) [56]
		Clinical placement	Perlman et al.(2017) [57]
		Unconventional Clinical placement	Moxham et al. (2016) [58]
		Unconventional Clinical placement	Cowley et al. (2016) [59]
		Clinical training	Ketola & Stein (2013) [60]
		Clinical training	Kragelund (2011) [61]
		Writing therapeutic letters	SmithBattle et al. (2010) [62]
		Clinical placement	Higgins & McCarthy (2005) [63]
	Learning through knowledge of lived experience	Experts by Experience teaching in the classroom	Happell et al. (2019) [64]
		Service user and carer involvement in students’ classroom	Unwin et al. (2018) [65]
		Service user involvement in the classroom	Rush (2008) [66]
Receiving knowledge passively	Learning through an explanatory theoretical approach	Education in general	Damsgaard et al. (2022) [67]
		Education in general	Farrington et al. (2020) [68]

#### Stage 5: collating, summarising and reporting the results

The final step in a scoping review is collating, summarising, and reporting the results. The aim of our study was to examine the body of knowledge on how the development of relational competence can be promoted and hampered in higher mental health education. In line with Thomas and Harden [69], and guided by the research questions, LSB and UEH conducted a thematic synthesis of the interventions described in the included studies. In stage one, LSB performed the coding based on the interventions. Two coding categories were developed: one capturing factors that promote the development of relational competence, and another capturing factors that may hinder it. In stage two, LSB and UEH examined similarities and differences among the codes and developed descriptive themes close to the primary data. In stage three, these descriptive themes were interpreted as analytical themes that mean “going beyond” the content of the included studies. The synthesis process was presented to the full research group where consensus was reached through discussion, leading to a revision of the results. Two analytical themes and six descriptive themes were identified (Table 4). An overview of the articles is presented in a simplified table (Table 5), while the themes are presented in a descriptive format [23].

**Table 5:** Description of the included studies

	Author (s) Year of publication	Country	Aims/purpose	Study population and sample size (if applicable)	Intervention type and comparator (if applicable)	Methodology	How outcomes are measured	Relational competence promoting factors identified	Relational competence hampering factors identified
1	Baisden & Gray 2020 [39]	USA	Learn about different voice hearing experiences, increase empathy and understanding and learn effective ways of helping people who hear distressing voices.	Multi-professional under-graduate students: nursing, psychology, and social work (*n* = 146) Completed surveys (*n* = 96)	Hearing Voices Simulation	Mixed methods design	Surveys (Pre and post) Narrative feedback in the surveys	x	
2	Bee, Watkins & Barnes 2023 [35]	USA	To examine effectiveness of the LifeRAFT model for training students in supportive counselling skills.	Senior-level psycho-logy students (*n* = 18)	LifeRAFT Training (role-playing activities)	Quantitative design Pre and post training	Client rating form Counsellor rating form Observer Rating form	x	
3	Campbell et al. 2017 [44]	USA	To investigate the use of the interactive video game “That Dragon, Cancer” as a tool to teach empathy	Third-year medical students Both surveys: (*n* = 45)	Video game	Quantitative design	Pre – and post survey A modified Jefferson Scales of Physician Empathy (JSPE)	x	
4	Chen et al. 2018 [40]	Australia	To evaluate the impact of participation in a mental health recovery camp on the clinical confidence of undergraduate nursing students in dealing with individuals with mental illness.	Nursing students (*n* = 20) Age ≤ 25: *n* = 8 Age > 26: *n* = 12	Mental health recovery-camp	Quantitative design Pretest/post-test design	Mental Health Nursing Clinical Confidence Scale (MHNCCS)	x	
5	Cowley et al. 2016 [59]	Denmark	To explore how psychiatric care is understood seen from a student perspective with focus on their personal competences and the educational interventions empowering processes for users’ personal and social recovery.	(*n* = 7) Nursing students (*n* = 4) Master nurses (*n* = 2) Master in applied philosophy student (*n* = 1)	Experiencing psychiatric care	Qualitative design	Semi-structured interviews		x
6	Damsgaard, et al. 2022 [67]	Irland	To explore mental health nursing students’ experiences of working with adolescents who are receiving inpatient treatment for an eating disorder.	Mental health nurse students (*n* = 4)	Work with adolescents with eating disorders	Qualitative design	Semi-structured interviews		x
7	Farrington et al. 2020 [68]	USA	To gain an understanding of nursing students’ personal perceptions of experiencing auditory hallucinations in a simulation and role-play activity before their first mental health clinical experience.	Nursing students (*n* = 40)	Hearing voices simulation and role-play	Qualitative design	Written surveys with four open-ended questions	x	
8	Fossen & Stoeckel 2016 [46]	UK	To evaluate practice-based critical incidents brought to reflective groups.	Mental health nursing students (*n* = 11) Facilitators (*n* = 2)	Facilitated reflective groups	Qualitative design	Interviews	x	
9	Gould & Masters 2004 [33]	Australia, Island, Ireland, Norway Finland, the Nether-lands	To explore nursing students’ experiences of Experts by Experience (EBE)-led mental health nursing education.	Nursing students 8 focus groups (*n* = 51)	EBE-led teaching	Qualitative design	Focus groups	x	
10	Happell et al. 2019 [64]	USA	Development and pilot-testing of 10 reflective journal prompts designed to stimulate reflection on emotional intelligence competencies.	Nursing students (*n* = 16)	Emotional Competence Reflective Prompts	Qualitative design	Students were writing responses to the prompts once a week	x	
11	Harrison & Fopma-Loy 2010 [32]	Israel	To examine whether emotional intelligence and empathy could be improved in the traditional classroom, employing experiential teaching modes.	Social work students (*n* = 165)	The course ‘Being a Therapist’	Quantitative design Pre- and post -questionnaires	The Schutte Self Report Emotional Intelligence Test (SSREIT) and the Interpersonal Reactivity Index (IRI)	x	
12	Hen & Goroshit 2011 [53]	Irland	To explore psychiatric student nurses’ experiences of having a mentor during their first practice placement with a view to identifying the strengths and weaknesses of the mentoring process.	Students under-taking a three-year diploma in nursing (psychiatry) (*n* = 6)	Mentorship during practice	Qualitative design	Semi-structured interviews	x	
13	Higgins & McCarthy 2005 [63]	Australia	This paper reports findings from a study of student nurses who received training and coaching in emotional intelligence (EI), a well-established correlate of resilience, just prior to undertaking a mental health or medical/surgical clinical placement.	A convenience sample of year 1 and year 3 nursing students (*N* = 42) Interviews (*n* = 12)	4-hour EI training workshop on the GENOS EI model prior to going on to placement.	Qualitative design	Semi-structured interviews	x	
14	Hurley et al. 2020 [38]	Norway	To examine short-term changes in occupational therapy students’ self-efficacy for using therapeutic modes, for recognizing clients’ interpersonal characteristics, and for managing interpersonal events. Factors associated with such changes were also examined.	Occupational therapy students (*n* = 89)	University-based and practice-based education. Intentional Relationship Model (IRM) workshops	Quantitative design	Three question-aires 2–3 weeks after a workshop and at 3 months’ follow-up	x	
15	Hussain et al. 2018 [43]	USA	To explore the learning experiences of students in a psychosocial nursing class that was infused with art, music, literature and film. Research question: What happens in a psychosocial nursing class when art, music, literature, and film are used to enhance learning about mental illness?	Nursing students (*n* = 23)	Psychosocial nursing course: Classroom activities that included art, music, literature, and film to enhance the student understanding of mental illness	Qualitative design	Questionnaires, observation, and focused interviews	x	
16	Jensen & Curtis 2008 [55]	Spain	To adapt the Psychiatric/Mental Health Clinical Placement (PMHCPS) to Spanish and examine its psychometric properties; to describe the attitudes of nursing students towards the mental health field.	Nursing students (*n* = 231)	Mental Health Practicum. Theoretical content and clinical placement	Quantitative design	Self-administered questionnaire	x	
17	Julia-Sanchis et al. 2020 [29]	USA	To explore the impact of a psychiatric clinical course in helping students learn to relate to distressed patients.	Nursing students (*n* = 67)	Psychiatric clinical course	Mixed methods design	Pre & post clinical questions	x	
18	Ketola & Stein 2013 [60]	Denmark	Investigation of nursing students’ learning process through clinical practise in mental health care. The ultimate aim was to optimize clinical learning.	Nursing students (*n* = 11)	Clinical placement	Qualitative design	Semi structured interviews and observation study	x	
19	Kragelund, L. 2011 [61]	UK	To improve communication skills training.	Nursing students (*n* = 520)	Role playing Simulation	Mixed methods design	Quantitative analyses 10-point Likert scale Free text comments in the survey	x	
20	Lewis et al. 2013 [49]	USA	A simulation design to enhance the learning of communication skills was developed.	Nursing students (*n* = 117)	Video-recorded standardized patient simulations	Qualitative design	Questionnaires Focus groups	x	
21	Miles et al. 2014 [48]	Australia	To determine whether stigma towards people with mental illness varied for undergraduate nursing students who attended a non-traditional clinical placement called Recovery Camp compared to students who attended a ‘typical’ mental health clinical placement.	Nursing students (*n* = 79) Recovery camp (*n* = 40) comparison (*n* = 39)	5 day-day immersive Recovery camp	Quantitative design Quasi-experimental design	Social Distance Scale (SDS) pre- and post-placement and at three-month follow-up	x	
22	Moxham et al. 2016 [58]	Australia	To examine whether differences in student self-determination was associated with the level of therapeutic relationship skills and clinical confidence within a mental health clinical placement.	Undergraduate nursing students (*n* = 279)	A mental health clinical placement in a non-traditional work-integrated learning (WiL) setting as Recovery camp	Quantitative design	The Work Task Motivation Scale, Therapeutic Relationship Scale, and the Mental Health Clinical Confidence Scale	x	
23	Patterson et al. 2023 [56]	USA	To examine the potential learning benefit (i.e., therapeutic relationships) in an innovative Recovery camp	Nursing students (*n* = 20)	5-day immersive clinical placement called Recovery camp	Qualitative design	Individual and focus groups interviews Observations Reflective journals	x	
24	Perlman et al. 2017 [57]	UK	To evaluate mental health nursing students’ experience of interpersonal skills assessment	Nursing students (*n* = 36)	Nominal group technique: A module combined didactic teaching of theoretical techniques with skills based practice of in clinical role play using video playback for group supervision.	Mixed methods design	Adapted form of Gaskins nominal group form. Thematic grouping	x	
25	Perry & Linsley 2006 [30]	USA	To evaluate nursing students’ satisfaction, self-confidence, and critical thinking related to the use of communication and assessment skills in conducting a mental status exam with standardized patients (SPs)	Nursing students (*n* = 112)	SPs simulating clinical encounters in psychiatric nursing.	Quantitative design	SP Observation Form	x	
26	Robinson- Smith et al. 2009 [54]	Spain	To examine the effect of the Memories escape room on nursing students’ stigma against severe mental illness (SMI).	Nursing students (*n* = 306)	Web-based Without Memories escape room	Quantitative design	A pre-post study questionnaire	x	
27	Rodriguez-Ferrer et al. 2022a [36]	Spain	To assess the use of educational escape rooms as a learning and awareness strategy on stigmatizing attitudes towards people with serious mental disorders in university students.	Nursing students (*n* = 44)	Web-based Without Memories escape room	Qualitative design	Open-ended survey	x	
28	Rodriguez-Ferrer et al. 2022b [37]	UK	To investigate the impact on student nurses’ practice and the underpinning mechanisms and contexts following service user involvement in the classroom	Nursing students (*n* = 26) Service users (*n* = 12)	Service user involvement in the classroom	Qualitative design	Individual and group interviews	x	
29	Rush, B. 2008 [66]	USA	To examine nursing students’ perspectives on the impact of writing therapeutic letters on their clinical learning and development of relational skills.	Nursing students (*n* = 74) Therapeutic letters (*n* = 140)	Therapeutic letters	Qualitative design	Focus groups Therapeutic letters	x	
30	Smith Battle et al. 2010 [62]	UK	To develop and evaluate a Talking Head video clip for use as part of a blended learning design to an introduction to applied psychology.	Nursing students (*n* = 100)	‘Talking Head’ video clips where authentic patients relate their experiences of illness and nursing care.	Mixed methods design	Focus groups Online demographic and attitudinal questionnaire and online quiz	x	
31	Snelgrove et al. 2016 [45]	USA	To evaluate the addition of a standardized patient (SP) simulation following the didactic lecture on schizophrenia on nursing student knowledge and competency of caring for a patient with this diagnosis.	Nursing students (*n* = 52)	Standardized patient (SP) simulation following the didactic lecture on schizophrenia	Quantitative design Quasi-experimental design	Visual Analog scale (VAS) scores	x	
32	Speeney et al. 2018 [42]	Norway	To explore the use of video as a learning tool in a post-bachelor program. Teaching care and cooperation with relatives of people with severe mental illness.	Nursing students (*n* = 4) Social work students (*n* = 1) Social educator students (*n* = 1)	Video of a family whose son is diagnosed with severe mental illness	Qualitative design	Focus groups	x	
33	Stiberg et al. 2012 [51]	USA	To investigate the impact of high-fidelity human simulation on nursing student anxiety prior to attending clinical and interacting with a mentally ill patient.	Nursing students (*n* = 44)	Simulation	Quantitative design	Demographic questionnaire (Pre and post)	x	
34	Szpak & Kameg 2013 [50]	Finland	To describe how psychology and medical students assess their own competency and skills (interpersonal and communication skills)	Psychology students (*n* = 183) Medical students (*n* = 126)	Training and role-play and communication skills assessment	Quantitative design	Semi-structured questionnaire (Pre and post)	x	
35	Tiuraniemi et al. 2011 [52]	USA	To determine if full immersion virtual reality simulation influenced nursing students’ anxiety levels and communication skills when caring for anxious patients.	Nursing students (*n* = 33)	Virtual reality simulation	Quantitative, quasi-experimental design	Demographics Survey Spielberger’s Short-Form State Anxiety Inventory Oxford Medical Simulation Communication Performance Analytics Dashboard	x	
36	Traister 2023 [34]	UK	To explore student learning experiences from SUAC involvement in courses at a university were evaluated for effects on student perceptions, knowledge, skills and practice.	Social welfare students (*n* = 8) Mental health nursing students (*n* = 6) Social welfare students (*n* = 8)	Involvement of service users and carers (SUAC’s)	Qualitative design	Focus group	x	
37	Unwin et al. 2018 [65]	USA	to determine whether a low- fidelity communication simulation laboratory would decrease nursing students’ perceived anxiety levels toward mental health patients and increase students’ perceived empathy, self-awareness, and active listening levels	Nursing students (*n* = 89)	A simulation intervention consisted of several components that included students’ pre-work, a lecture, a case study session, and the role-play SBLE.	Quantitative quasi- experimental Pretest – post-test design	The State–Trait Anxiety Inventory (STAI, Form Y-1) The Jefferson Scale of Empathy–Health Professions Student Version (JSE-HPS) The Situational Self-Awareness Scale (SSAS)	x	
38	Waugh et al. 2014 [31]	UK	To evaluate the effectiveness of Action Learning Sets (ALS) as a teaching and learning strategy within a Pre-registration Mental Health Nursing Programme from the perspective of the students and facilitators who are mental health lecturers	Nursing students (*n* = 10) Lecturers (*n* = 7)	Introduction of ALS in Mental Health Nursing Programme	Qualitative design	Focus groups	x	
39	Webster 2014 [47]	USA	To examine whether there were differences in nursing student empathy after a specific creative teaching strategy was implemented with some (comparison group) and not with others (control group).	Senior bacca-laureate nursing Students (*n* = 73) Comparation group (*n* = 29) Control-Group (*n* = 44)	Standardized patient experiences (SPEs) as a teaching modality	Mixed methods: Pretest-post-test quasi-experimental design Interviews, field observation, and student creative reflective assignments	IRI item self-report tool and interviews.	x	
40	Witt et al. 2018 [41]	USA	To examine the effect of SP simulation on a simulated home environment to promote senior nursing students’ ability to assess and communicate with patients with mental health conditions	Nursing students (*n* = 32)	Simulation development	Quantitative design Randomized trial	A six-item author-developed demographic survey A ten-item multiple-choice National Council Licensure Examination- style pretest/posttest	x	

## Results

**Fig. 1:**
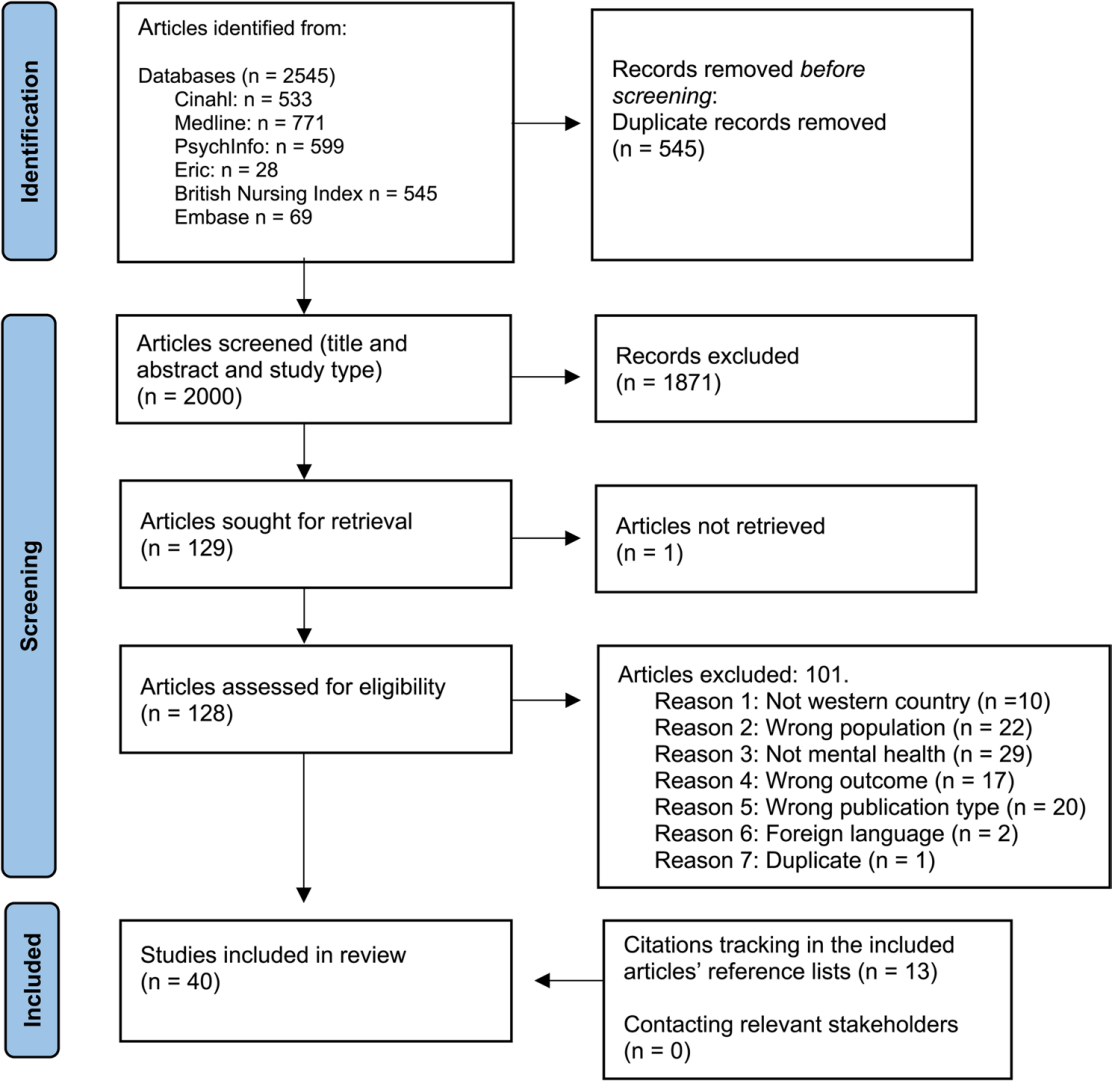
Prisma flow diagram

The forty scientific studies included in this review (Fig. 1) employed qualitative methods (*n* = 18), quantitative methods (*n* = 16), and mixed methods (*n* = 6). Student voices are represented in all included studies through individual interviews, focus group interviews, surveys, and narrative feedback in surveys. Descriptions of included studies are reported in Table 5.

A synthesis conducted to systematise the studies’ (*n* = 40) results shows both promoting and hampering interventions regarding the development of relational competence in the mental health education of healthcare professionals. Learning interventions were organised into two themes. The first, *individual active participation*, describes promoting interventions and includes the sub-themes *mapping training needs* (*n* = 2), *learning through reflection* (*n* = 3), *learning through active engagement in the classroom* (*n* = 22), *learning in real-life situations* (*n* = 8), and *learning through knowledge of lived experience* (*n* = 3). The second theme, *receiving knowledge passively*, describes hampering factors, which includes the sub-theme *learning through an explanatory theoretical approach* (*n* = 2).

### Individual active participation

An overall understanding of the interventions described in the literature to promote relational competence is that they involve the individual student’s active participation, which refers to students’ active engagement and involvement. To facilitate individual active participation, a starting point is identifying students’ competencies that require improvement by *mapping training needs*. Teaching can focus on these factors by identifying the areas of student attitudes and competencies that require improvement [29, 30]. For access to student attitudes towards people with mental health problems, students can participate in validated mental health clinical placement surveys [29]. By way of the so-called nominal group technique, students can participate in groups generating ideas about the teaching and assessment of interpersonal skills [30]. By teachers engaging and collaborating with the students, students may be helped to take an active role in the development of relational skills.

Three studies describe interventions aimed solely at *learning through reflection*, although several studies in this review describe reflection as essential to accomplishing the intervention. Reflection is important for the development of relational competence and can be stimulated through various activities. Harrison and Fopma-Loy [32] imply that progressive journal prompts are useful tools for introducing and stimulating reflection on emotional intelligence competencies in nursing students. Combining individual written journal prompts and group discussion may thus provide an assessment of students’ emotional intelligence competencies. When desired competencies are targeted, the reflections associated with the journal prompts help students draw further connections between their journalling and needed changes. According to Waugh et al. [31], action learning is a strategy that supports the development of relational competence. They describe action learning as a process of reflection and learning that happens while working with cases from real life, with the support of a group of students and lecturers. The reflections seek a new understanding of a situation and encourage the participants to take new actions. Through action learning, students engage in and develop reflective enquiry [31].

Gould and Masters [33] found that reflective groups allowed the sharing of various challenging thoughts and experiences from practice and contributed alternative ideas and understandings. However, the reflective groups’ facilitation style was seen as crucial, as the facilitator seemed to influence the atmosphere within the group. Constructive group dynamics, where students are confident enough to share thoughts and experiences, are reported as important for productive group reflections [33].

*Individual active participation* is facilitated by *learning through engagement in the classroom.* Various classroom activities stimulate engagement. Classroom activities which include art, music, literature, online games, and film to enhance students’ understanding of mental illness have been found to be catchy and provide a deeper understanding, develop sensitivity and attitudes, and increase cultural awareness, as well as developing self-understanding and empathy [45, 51, 55]. Snelgrove et al. [45] report that a person with mental health challenges’ narrative in teaching holds greater persuasive power in education than does information found in textbooks, probably because the narrative captures the students’ attention due to the narrator conveying personal experiences that are challenging to convey through text alone. Classroom activities focusing on emotional intelligence training and coaching have been shown to equip students with higher relational competence [38, 53]. Hurley et al. [38] demonstrate that emotional intelligence training, delivered through active classroom engagement, fosters resilience, empathy, and compassion among students. It also enhances their ability to respond constructively to individuals with mental health challenges and strengthens their non-technical workplace skills.

Interactive video games are an activity that also promotes individual engagement in the classroom. Interactive video games developed to teach empathy have been shown to be useful for improving empathy in innovative curricula. In their study of teaching empathy, Chen et al. [40] confirmed that most students in the study concurred that acquiring empathy through video games was more favourable and efficient than traditional teaching methods. Rodríguez-Ferrer et al. [37] found online video games a motivating learning strategy where students participate and engage actively in the curricular content. Their online game focused on stigmatising attitudes, and the students became more sensitised and developed a greater understanding of people with serious mental disorders when playing this game [36].

Simulation is a classroom activity requiring a high level of student engagement. Simulation imitates a real-world situation, making it possible for students to experience interaction in clinical situations by practising in a safe learning space, with no consequences for real persons. The students are placed in imitational situations, either role-play with actors trained to act as persons with mental health challenges or role-play with students or teachers as actors, in real life or virtually. Simulation has proved to efficiently increase participants’ empathy [39, 46] and understanding of the interprofessional team [39], as well as their communication and interpersonal skills [35, 41, 43, 47–49, 52, 54]. The study by Witt et al. [41] found that simulation improved communication skills in the short term but not in the longer term, which they indicate is consistent with previous research. Simulation is indicated as an effective method for training students in helping skills [42, 44, 46], raising awareness of attitudes [36, 46], and decreasing their anxiety prior to communicating with mentally ill persons [35, 50].

Traister et al. [34] found students’ communication scores increased somewhat after a virtual-reality simulation. However, the results indicated a lack of communication skills both before and after the intervention, which the researchers indicated could reveal a need for more communication-based experiences in education.

However, facilitator competence is deemed a key factor in ensuring good outcomes [49]. Student-to-student mentoring in a simulation context is considered valuable for teaching interpersonal skills. Students appreciate sharing valuable clinical experiences with their peers. The study by Miles et al. [48] found that the peer mentorship approach affected how one sees oneself; it provided an opportunity for self-evaluation and was significant for practice.

*Learning through knowledge of lived experience* is shown to engage students’ active participation in dialogue and self-examination [66]. When interacting with persons with mental health challenges in the classroom, the students heard about first-person experiences, which strongly engaged them and helped them to understand these individuals as persons rather than as a diagnosis. This developed the students’ respect and their insight into needing to look beyond the obvious and reflect more. It also helped them to understand how they might forge empathic relationships with persons with mental health challenges and become more capable of passing on hope and thus supporting their recovery processes [64–66]. Learning from lived experience in the classroom also prepared students for realistic challenges in practice [65]. Unlike a traditional lecturer, a person with mental health challenges represents the “reality” that engages the students emotionally and serves as a reminder of how persons with mental health challenges would like to be treated. Awareness of power issues in relationships grows in students when a person with mental health challenges takes on the role of helping the students in the classroom and the students are the learners [66]. Involving persons with mental health challenges in the classroom has been shown to help catalyse transformative learning, linking theory to practice and enhancing relational competence [64–66].

Experience gathered through clinical placements provide students with *learning in real-life situations* that support the development of relational competence [57, 59, 60]. Many students lack previous experience of engaging with people with mental health problems and thus feeling anxious or afraid and less confident about meeting them. Clinical placement allows students to gain knowledge and insight into what it means to live with mental illness [59]. Students report less prejudicial feelings towards and less fear of violence from persons with mental health challenges after completing clinical placements [60]. Clinical placements provide students with collaborative and immersive experiences that allow for the development of relational competence [57]. Student motivation in a clinical placement setting is important. Patterson et al. [56] found that students with higher motivation conveyed significantly higher relational competence after clinical placements, and a non-traditional learning environment may enhance student motivation. A non-traditional clinical placement is a placement outside of a typical inpatient setting. Moxham et al. [58] found that students attending a non-traditional clinical placement called Recovery Camp (an Australian “bushcamp” setting) reported significant reductions in stigma towards people with mental illness over time than students attending a typical clinical placement. The presence of a mentor in a student’s clinical placement significantly supports the student’s learning as the mentor provides non-critical, supportive feedback and promotes reflection on practice. Mentors play a key role in guiding and supporting students, and the student-mentor relationship can help develop relational competence [63]. Learning in clinical placements can be enhanced by students and mentors together identifying unconscious learning opportunities and becoming consciously aware of typical learning situations [61].

Writing therapeutic letters has been shown to promote student reflection on improving helping relationships, cultivating these skills, and developing relational competence [62]. A therapeutic letter is written by the student and sent to the patient or family between their encounters, functioning as a tool for supporting the receiver, building trust, and strengthening thoughts discussed in previous clinical encounters. Researchers have considered this a developing activity. However, if students approached writing therapeutic letters as an instrumental activity, they offered little reflection on the process without engaging with the patient, and relational competence was not cultivated [62].

Overall, individual active participation in developing relational competence involves an engaged approach to enhancing the skills and attitudes necessary for therapeutic relationships with persons with mental health challenges. Incorporating these interventions in mental health education of healthcare professionals will enable students to develop their relational competence.

### Receiving knowledge passively

Two of the included studies describe hampering interventions regarding the development of relational competence in mental health education of healthcare professionals as an explanatory theoretical approach [67, 68]. An explanatory theoretical approach makes it difficult to develop students’ personal competencies during education in practice and theory. A traditional biomedical education is dominated by “knowledge about” and lacks a focus on values, ethics, and attitudes. Consequently, if not focusing on person-centered and values-based care, this approach does not prepare students for meeting persons with mental health challenges relationally. Students learn how to observe them and treat them based on a diagnosis, without focusing on the person, the person’s life, or how to interact with them [67, 68]. Focusing principally on theoretical knowledge will not prepare students to engage therapeutically and will hamper the development of relational competence. Students learn relational skills by observing qualified health professionals [57, 63]. The development of relational competence may be hampered when students experience exclusion during clinical placements [68]. Exclusion during clinical placements not only limits students’ access to relational experiences but also models behaviors that contradict relational competence. This exclusion fosters a passive learning environment, where students receive knowledge without engaging in the interpersonal processes necessary for developing their relational competence. Overall, receiving knowledge passively does not promote the development of relational competence, as relational competence is difficult to learn solely from the distance of “knowledge about”.

## Discussion

This scoping review explores how the development of relational competence can be promoted and hampered in mental health education of healthcare professionals and identifies knowledge gaps in this field. Learning interventions were organised into two themes. The first, individual active participation, describing promoting interventions, which includes the subthemes mapping training needs, learning through reflection, learning through active engagement in the classroom, learning in real-life situations, and learning through knowledge of lived experience. The second theme, receiving knowledge passively, describes hampering factors, which include the sub-theme of learning through an explanatory theoretical approach. In this discussion, we wish to reflect on our results in connection with Kolb’s learning model [22], which provides a valuable framework for understanding how relational competence can be developed in mental health education. This model will be used to discuss the results and identify knowledge gaps in this field.

### Various learning strategies

Our results highlight the importance of facilitating students’ individual active participation in developing relational competence and how various approaches are suitable in this regard. However, the interventions used to promote students’ relational competence are different, so it is impossible to determine which intervention is suitable in different situations. According to Kolb [22], it is important to activate students in different ways to ensure that different types of learning are facilitated and that students achieve learning through observation, reflection, and task performance as part of the learning experience. This may accommodate the wide range of students and their preferred teaching methods rather than focusing primarily on theory-oriented learning. This may also enhance student’s capability for developing and maintaining therapeutic relationships with persons with mental health challenges, as it may require a dynamic and empathic approach requiring both personal and theoretical skills [70].

For in-depth learning, students in mental health education of healthcare professionals must understand the various terms, perspectives, histories, and contexts linked to the persons with mental health challenges they shall help. Thus, assimilative learning must be the basis for a more advanced form of learning [22]. Theoretical knowledge about important elements of relational competence will help students see connections and put these into a meaningful context [71]. Furthermore, students must become aware of how knowledge of relational competence can be applied in different situations, where this knowledge is organised holistically and linked to practice through accommodative learning. For in-depth knowledge, factual knowledge must be anchored in one’s own experiences in the field of practice [22]. It is therefore important to combine theoretical background and experience-based teaching strategies to develop relational competence [71]. In combination, theoretical knowledge and relational understanding can provide students with a good starting point and competence for future work with people with mental health challenges. Reflection helps students develop their own experiences into professional knowledge [72] about establishing therapeutic relationships with persons with mental health challenges.

Active engagement helps students translate theoretical concepts into therapeutic relational practices [22]. However, Damsgaard et al. [67] report that an explanatory theoretical approach still seems to dominate psychiatric healthcare education. Based on biomedical understanding and the acquisition of what is considered relevant knowledge, exploration and focus on the development of therapeutic relationships can be de-prioritised. “Knowledge about” is often based on biomedical facts largely considered objective. Passive learning often involves memorising the right answer without a deeper understanding of how knowledge applies in real clinical situations. This may lead to practice based on prejudice, which is described as ineffective for persons with mental health challenges’ recovery [10]. Through accommodative learning, understanding and interpretation-based knowledge are promoted [22]. By engaging students, the teacher can help them to understand and appreciate complex topics that do not necessarily have a right or wrong answer, such as by encouraging students to let go of the need to be right. Teachers can encourage students to be aware of their own and others’ ways of thinking rather than focusing unilaterally on opinions per se. This will enable them to reflect on, react to, and understand different situations professionally [5] and help them move away from black-and-white thinking by seeing different facets of experiences, opinions, and experiences [73].

### Subjective learning experiences

Relational competence involves navigating complex interpersonal dynamics and thus requires practical skills and the ability to adapt theoretical knowledge to diverse interpersonal scenarios [22]. Without active engagement, students may struggle to translate theoretical concepts into effective relational practices. An explanatory theoretical approach entailing assimilative learning is important for developing knowledge, but to achieve competence in establishing therapeutic relationships, mental health education of healthcare professionals must also train students in the development of relational competencies such as empathy, active listening, and communication [15]. When empowering students to master highly emotionally charged situations, their learning process, engagement, and subjective learning experiences will be of far greater importance than in traditional theory-based learning. Developing relational competence requires self-awareness and an understanding of one’s own biases, values, and communication style. Passive learners may not engage in reflective practices that promote self-awareness. Without active self-reflection, individuals may be less attuned to their emotional reactions and less capable of managing them in relational contexts [22]. Students’ reflections on topics such as who they are, how they affect the patient, and how to create trust are therefore important to facilitate learning. Creating a safe climate becomes essential so that students can openly explore themselves and their empathic abilities and their listening, questioning, and mirroring skills.

Mentors modelling good relational practice during clinical placement are vital for students’ learning outcomes [71]. To promote relational competence in mental health education of healthcare professionals, the teacher/mentor must act as a role model by creating an empathetic climate in learning situations, focusing on improving emotional self-regulation, and leading mindfulness-based interventions [53]. By creating a safe environment, different points of view can be brought out and respect for everyone maintained. Teachers must use the opportunity to identify and regulate emotions that arise in teaching. Clear guidelines for respectful dialogue on sensitive topics must underpin the handling of the various conflicts and differences that arise. The teacher models for the students the importance of respectful behaviour, emphasising dialogue about different perspectives of understanding. The teacher can show that he/she “senses” affect (e.g. facial expressions) by putting it into words and interpreting and communicating how it is experienced. Teachers further emphasise that emotional reactions can be challenging but are normal in learning and must be recognised and managed [73]. By modelling, the teacher/mentor creates space for transformative learning [22, 71] and helps students to understand and normalise the emotional reactions they have in real situations and to change how they respond. Thus, teachers must have solid knowledge and personal skills in relational competence. It is fundamental to be able to link theory to experience-based teaching strategies, model relational competence for students, and help them construct meaning. Without this competence, the teacher/mentor will not be able to facilitate pedagogical approaches that promote relational competence.

### Knowledge gaps related to development of relational competence in mental health education of healthcare professionals

Addressing knowledge gaps is crucial for advancing the understanding and implementation of strategies to develop relational competence in higher mental healthcare education. Accommodative learning assumes that students already have an understanding that can be broken down and restructured [22]. First-year students do not have the same foundation of practical experience as advanced students. One must master knowledge at a lower level before processing knowledge at a higher level [74]. We have not found research-based knowledge about how students lacking or with little experience can best become connected to accommodative learning. This focus is needed in future research.

Online teaching is a growing trend in higher education. In online settings, teachers and students do not meet physically, which affects the psychological atmosphere and learning engagement. This influences learning in various ways [75]. None of the studies included in this review addressed these issues. How to work pedagogically to promote relational competence under these conditions needs to be explored [73].

A range of interventions have been identified for developing relational competence. Whether there are specific pedagogical strategies to promote relational competence needs further exploration.

## Limitations and strengths of the review

Our scoping review mirrors the complexity and heterogeneity of the scientific literature on developing relational competence [23]. Descriptions of relational competence vary in the literature; a clear definition could contribute to stricter searches that might yield more comparable results.

A strength of this review is the comprehensive systematic search strategy conducted by a qualified librarian in collaboration with two researchers (LSB and UEH) and the blinded screening process in Rayyan carried out by all researchers in pairs. According to Arksey and O’Malley [25], quality assessment of included publications is not necessary, but we consider our narrative description of their quality to be a strength. The data charting, collating and summarizing process involved two researchers initially (LSB and UEH) and was later validated by the whole group.

Inclusion of publications from non-indexed journals, book chapters, and grey literature might have broadened our findings. However, as the quality of this literature can be challenging to assess, publications with shortcomings could have influenced the results. Additionally, the exclusion of non-Western countries represents a limitation, as it may restrict the generalizability of the findings across diverse cultural and healthcare contexts.

## Conclusion

Interventions focusing on students’ individual active participation seem to promote the development of relational competence. This review of scientific literature has identified various interventions facilitating students’ engagement and involvement, including mapping training needs, learning through reflection, learning through active engagement in the classroom, learning in real-life situations, and learning through knowledge of lived experience. Receiving knowledge passively without practical application and experiential learning is insufficient for developing the relational competence required for high-quality practice in mental health settings. Learning solely through an explanatory theoretical approach is found to hinder the development of relational competence.

### Implications for practice and further research

While various interventions have been identified to develop relational competence, there is a dearth of research exploring specific pedagogical strategies for promoting it. This highlights the need for further investigation into effective teaching methods that can enhance relational competence among students in mental health education. More knowledge is needed on strategies to facilitate the connection between accommodative learning and students lacking practical experience. Further investigation is also needed into pedagogical approaches that can promote relational competence in online settings. Educational programmes should prioritise active engagement strategies to better prepare students for relational interactions with persons with mental health challenges.

## Data Availability

Authors can confirm that all relevant data are included in the article.
